# A Case of Supposed Indigenous Leprosy

**Published:** 1890-11-08

**Authors:** 


### THE PRACTITIONER'S MIRROR AND RETROSPECT.

[*The Editor will be glad to receive offers of co-operation and contributions from members of the profession. There is no doubt that much valuable material full of interest and instruction is at present lost to science. This is largely so in the case of* (1) *the younger and less-known members of the hospital staff* (2) *those connected with cottage hospitals, and* (3) *the great body of medical practitioners not attached to any institution. The co-operation of all is cordially invited to enable the Editor to make* THE HOSPITAL *of the utmost possible use and value to the profession. Specialists willing to review books on their special subjects are invited to communicate this fact at once. All letters should be addressed to* THE EDITOR, THE LODGE, PORCHESTER SQUARE, LONDON, W.]

### THE PATHOLOGICAL SOCIETY OF LONDON.

### A CASE OF SUPPOSED INDIGENOUS LEPROSY.

At the meeting on Tuesday November 4th, W. H. Dickinson, M.D., president in the chair, Dr. Phineas Abraham showed specimens and drawings from a patient who created considerable attention a short time ago. In the month of May last paragraphs appeared in the daily papers stating that there was a case of undoubted leprosy in Warwickshire. Dr. Abraham was requested to visit the case and to advise what should be done with the supposed leper as there was so much alarm in the neighbourhood. He found the patient a woman aged 69 years, lying in bed apparently in great pain and without any proper nursing. The face, body, and limbs were greatly emaciated, the left arm exhibited a large fungating growth at the region of the elbow joint above which the arm was much swollen and oedematous, the skin of the forearm and hand was thick and brawny, scarred in several places and mottled with a dark brown discolouration. The fingers were flexed, some of the terminal phalanges were gangrenous and the nails overgrown and clawlike. The ungual phalanx of the little finger had been spontaneously amputated through the process of ulceration. There was some power of movement of the fingers, but there was no well marked anæsthesia. The other extremities and the skin over the rest of the body showed no spots, patches, or any sign of disease. Every other part of the body, with the exception of this arm and hand, was (and according to the patient had always been) perfectly healthy. She stated that about thirty years ago she noticed a growth like a bean on the forearm, the skin became scaly and thick around, and this condition gradually extended towards the hand and elbow. The swelling of the limb was comparatively recent, and the cauliflower mass about the elbow had only appeared about two months. Her general health had always been good and there was nothing of importance in the family history.

She was born at Coventry, and had never been out of Warwickshire; was the wife of a labourer, who at the time used to go about the country selling rags and bones. She refused to go into a hospital, and died a few weeks afterwards in her own home. No post-mortem was permitted, but Mr. Pitt, under whose care she was, excised the forearm and arm, which was now exhibited to the society, together with the drawing taken the day after death. A series of microscopical specimens were shown from different parts. No leprosy bacilli could be found. The sections showed the tumour at the elbow to be a rapidly infiltrating epitheliomatous neoplasm. There were some large protoplasmic masses both within the cell nests and also outside, which were somewhat similar in appearance to "Psorosperms." Sections from the circumscribed patch of skin of the forearm also indicated just commencing epithelioma, as did sections taken from the separated ungual phalanx. In some of these sections giant cells were seen beneath the epidermis, and these were surrounded by granulomatous tissue, as is seen in lupus. This description tended to support Mr. Hutchinson's view of the case, namely, that it commenced as a superficial lupoid ulceration, scarring over the spot first attacked and spreading at the edges, and that the epithelioma had been a secondary development. Apart from the pathological interest of the specimen, the case appeared to the author to be worthy of note from another point of view, namely, it was no doubt in a great measure due to the clawed appearance of the hand, the spontaneous loss of one of the phalanges, and the long-continued desquamative condition of the limb that the idea arose of the case being one of leprosy. Had the patient succumbed a few weeks earlier, we should probably have had to quote her in future as a possible case of "indigenous leprosy," and our etioglogical inquiries concerning that disease would be still further confused by another doubtful recorded case.

Dr. Colcott Fox concurred with the view expressed, that the case was one of tubercular disease of the limb, with secondary epitheliomatous growth. Leprosy was a symetrical and bilateral disease, and not so localised as this one. The clinical history was also in favour of it being tubercular, so also the giant form of the limb, which was characteristic of lupus and due to lymphangitis. Some few cases of supposed indigenous leprosy had been recorded in England, but many of these when investigated had turned out to be "granuloma fungoides," and other diseases, but not leprosy. In fact he very much doubted if leprosy ever had been indigenous in this country. Several asylums had been established in olden times, but in many cases directly the details of the cases were produced, they were found to be cases of lupus and syphilis.

Dr. Samuel Wilks could not help believing that there were cases of indigenous leprosy in this country, and instanced some models of the disease which were in the Guy's Hospital Museum, which were taken from a man who had never been out of London. The case was under the care of Addison, who at the time was not quite certain of the true nature of the case, and called it "spurious leprosy." The man died from ulceration of the larynx with all the appearances of that condition which is known to occur in leprosy, and the models strongly support this opinion.

Mr. Targett proposed that the specimen should be referred to the "Morbid Growths Committee" to report upon. He had examined the ulnar nerve, and in some parts found some thickening of its sheath.

Mr. R. Williams said he had never seen an epitheliomatous ulcer at all like this one. He considered that the only criterion between epitheliomatous ulcers and chronic ulcers was the continuous growth of the cells in the former.

Mr. Gordon Brodie considered the appearances of the sections of the growth, especially the nature of the cell-nests were those of epithelioma.

Dr. Dickinson said that if we believed in the traditions of our forefathers, we must certainly acknowledge that leprosy was indigenous in this country, and referred to the leper windows in some churches; but these were only in very old churches, and usually in those by the seaside. He would suggest that the specimen in question be referred to a special committee, consisting of Mr. Watson Cheyne, Dr. Thin, and Dr. Crookshank.

